# Impact of the COVID-19 pandemic on quality of life and emotional wellbeing in patients with bone metastases treated with radiotherapy: a prospective cohort study

**DOI:** 10.1007/s10585-021-10079-x

**Published:** 2021-02-25

**Authors:** M. M. T. J. Bartels, R. Gal, J. M. van der Velden, J. J. C. Verhoeff, J. J. Verlaan, H. M. Verkooijen

**Affiliations:** 1 Division of Imaging and Oncology, Trial Office, University Medical Center Utrecht, Heidelberglaan 100, Internal mail Q.00.311, 3584 CX Utrecht, The Netherlands; 2 Division of Imaging and Oncology, Department of Radiotherapy, University Medical Center Utrecht, Heidelberglaan 100, 3584 CX Utrecht, The Netherlands; 3 Division of Surgical Specialties, Department of Orthopedic Surgery, University Medical Center Utrecht, Heidelberglaan 100, 3584 CX Utrecht, The Netherlands

**Keywords:** COVID-19, Pandemic, Quality of life, Patient reported outcomes, Metastasis, Radiation oncology, Palliative care, Social isolation, PRESENT-study

## Abstract

Implementation of COVID-19 measures may have induced concerns about access and quality of health care for cancer patients with bone metastases, and it may have affected their quality of life. In this study, we evaluated the effect of the first COVID-19 lockdown on quality of life and emotional functioning of patients with stage IV cancer treated for painful bone metastases in the UMC Utrecht, the Netherlands. A COVID-19 specific questionnaire was sent to active participants in the Prospective Evaluation of interventional StudiEs on boNe meTastases (PRESENT) cohort, consisting of patients irradiated for metastatic bone disease. Patient reported outcomes (PROs) were compared with the last two PROs collected within the PRESENT cohort before the COVID-19 lockdown in the Netherlands on the 16th of March. For the 169 (53%) responders, median age at start of lockdown was 68 years (range 38–92) and 62% were male. Patients reported a statistically significant decrease in emotional functioning (83.6 to 79.2, P = 0.004) and in general quality of life score during the COVID-19 lockdown (72.4 to 68.7, P = 0.007). A steep increase in feeling isolated was reported (18% before and 67% during lockdown). This study has shown a strong increase in the experience of isolation and a decrease of emotional functioning and general quality of life during the COVID-19 lockdown in cancer patients with bone metastases. Due to the nature of the treatment of this patient population, efforts should be made to minimize these changes during future lockdowns.

## Introduction

On March 16th 2020, the first official lockdown was announced in the Netherlands as a reaction on the outbreak of the SARS CoV-2 pandemic. Other emergency measures taken included the use of medical and non-medical facemasks, social distancing and targeted quarantine. [1] The lockdown influenced many aspects of oncology care. [2–7] To accommodate the increasing pressure on the health care system in The Netherlands, elective health care was postponed as much as possible. This included postponement of most oncologic care to minimize infection risk, as cancer patients are considered a high-risk patient population that would suffer severe complications when infected with SARS-CoV-2. [3, 4] Therefore, national and local oncological health care protocols were modified to minimise the risk of transmission of the virus and maximize capacity for COVID-19 care. [8]

This unprecedented situation is expected to have impacted the life of many patients with metastasized cancer: implementation of the COVID-19 measures may have induced concerns about their (access) to treatment and continuity of health care. [9] In addition, measures of social distancing may have incapacitated caregiver support networks and informal care schedules. [10] Since this palliative patient population may not have the opportunity to catch up on lost time after the pandemic has stabilized, mental health and emotional functioning may have been seriously affected as well. In this study, we evaluated the effect of societal COVID-19 measures on changes in quality of life and emotional functioning of patients with metastatic bone disease.

## Methods

### Study design and participants

The current study was conducted within the Prospective Evaluation of interventional StudiEs on boNe meTastases (PRESENT) cohort. [11] The PRESENT cohort includes patients with bone metastases, referred to the Department of Radiation Oncology of the University Medical Center Utrecht (UMCU) in the Netherlands. Patients are invited to participate in PRESENT prior to their appointment with the radiation oncologist. Patients consented to longitudinal collection of clinical data through medical records, and for receiving questionnaires at regular intervals during and after radiation treatment. The questionnaires consisted of: Brief Pain Inventory (BPI), European Organisation for Research and Treatment of Cancer quality of life questionnaires (EORTC-C15-PAL and EORTC-BM22) and the EuroQoL five-dimensional instrument of health-related quality of life (EQ5D-3L). [11–14] The PRESENT-study was approved by the Medical Ethics Committee of the UMCU (NL49273.041.14, METC 13/261) and was registered on clinicaltrials.gov (NCT02356497). For the current study, an additional COVID-19 specific questionnaire was sent out to active PRESENT cohort patients who had given informed consent to receive quality of life questionnaires. This questionnaire was approved as an addendum to the PRESENT study by the Medical Ethics Committee of the UMCU, and consisted of selected questions from the BPI, EORTC-C15-PAL and EORTC-BM22 questionnaires, as well as questions developed by the study team to evaluate the impact of COVID-19 measures on health care.

### Data collection

On the 7th of April 2020, the additional online questionnaire was sent out to active PRESENT cohort participants, shortly after the start of the national COVID-19 (partial and “intelligent”) lockdown on the 16th of March. Patient reported outcomes (PROs) within two years before the start and during the COVID-19 lockdown (either collected with the specific COVID-19 questionnaire or through regular follow up cohort questionnaires) from individual patients were included. Questionnaires filled in during lockdown where defined as ‘during lockdown’(DL). The most recent questionnaire before the start of lockdown was defined as ‘before lockdown-1’(BL-1); the questionnaire that was filled out previous to that one was defined as ‘before lockdown-2’(BL-2). If no questionnaire was completed within two years before the start of the COVID-19 lockdown, the pertaining patient was excluded from analysis.

### Statistical analysis

Proportions, frequencies and means (M) with standard deviations (SD) or medians with interquartile ranges (IQR) were used to describe patient and tumor characteristics. Differences in PROs before and during COVID-19 lockdown were analysed using a paired *T*-test. All reported p values were two-sided and p-values < 0.05 were considered statistically significant. Statistical analyses were performed with the use of IBM Statistical Package for Social Sciences (SPSS) software, version 25 (IBM Corp, Armonk, NY).

## Results

**Fig. 1:**
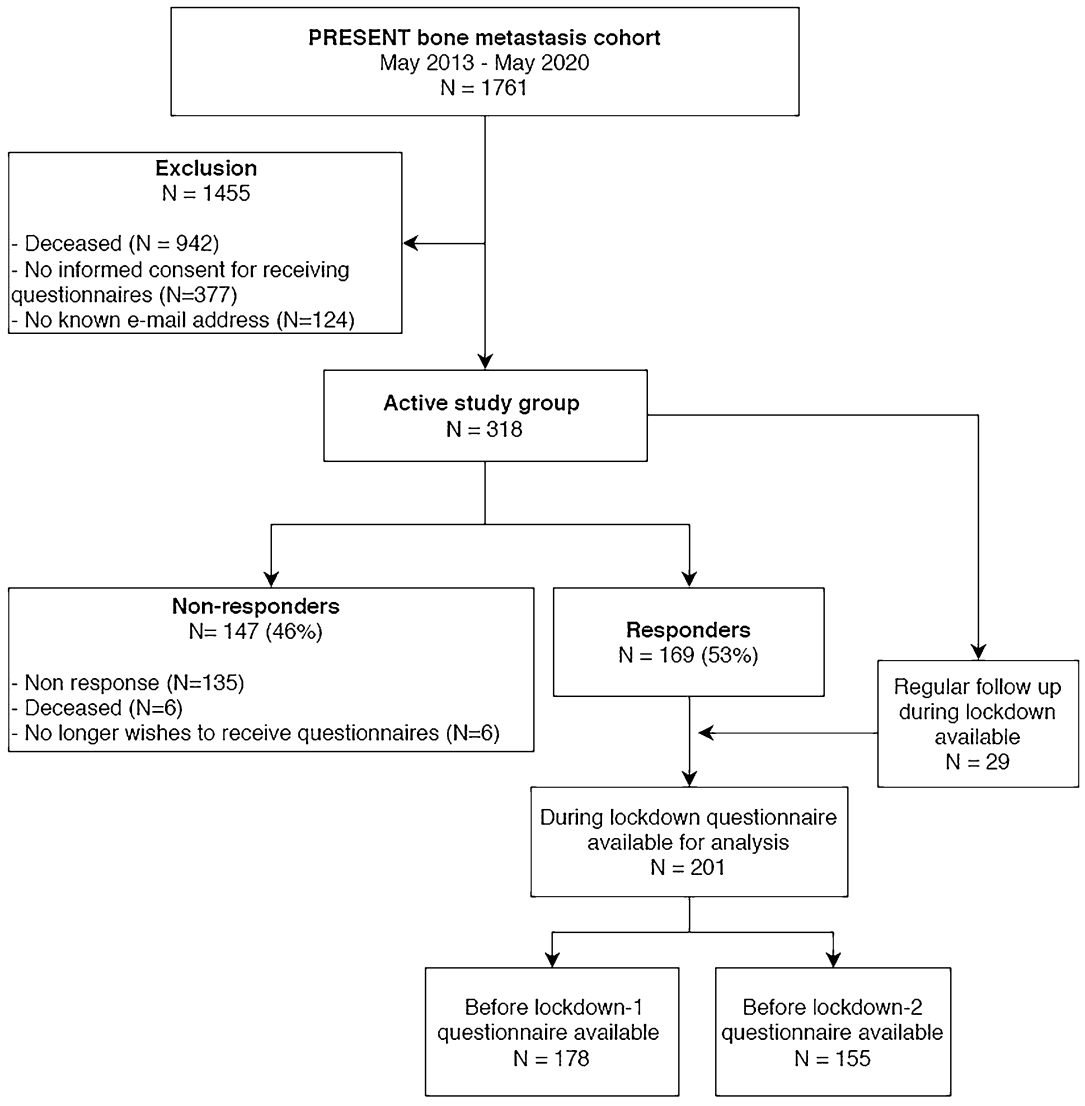
Flowchart of study inclusion. Flowchart of study inclusion. 169 responded to the COVID specific questionnaire. For analysis of the PROM’s regular follow up questionnaires were added to the COVID-specific questionnaires. This resulted in 201 patients, of which 178 were available for comparison between before lockdown-1 and during lockdown, and 155 for comparison between before lockdown-2 and during lockdown

Between May 2013 and May 2020, 1761 patients were included in PRESENT, 819 of whom were still active participants. The extra COVID-19 questionnaire was sent to the 318 active PRESENT participants who agreed to receive questionnaires. Of these patients 169 (53%) completed the COVID-19 questionnaire (Fig. 1). Median age at the start of the lockdown was 68 years (range 38–92), 62% of the patients were male and 88% lived together with their partner and/or children (Table 1). Patient and tumor characteristics were comparable between responders and the overall PRESENT cohort as described in the prospective evaluation of the cohort [11]. The median time between the start of lockdown and the two most recent questionnaires before the start of lockdown was 3 and 8 months respectively.

**Table 1:** Baseline characteristics of responders and the PRESENT-cohort

	Responders (n = 169)	PRESENT-cohort^a^ (n = 432)
Gender		
Male (%)	104 (62)	255 (56)
Age at inclusion (years)		
Median (range)	66 (33–92)	67 (28–90)
Primary cancer site (%)		
Prostate	54 (35)	127 (29)
Breast	40 (26)	97 (23)
Lung	28 (18)	97 (23)
Other	34 (22)	111 (25)
Non-bone metastases (%)		
Visceral	64 (38)	170 (39)
Brain	2 (1)	6 (1)
Radiation treatment		
8 Gy; 1 × 8 Gy	72 (43)	290 (67)
30 Gy; 10 × 3 Gy	46 (27)	72 (17)
Other	51 (39)	70 (16)
Age at time of start lockdown (years)		
Median (range)	68 (38–92)	N/A
Time between lockdown date and questionnaire (median)		
Before lockdown-1	3 months	N/A
Before lockdown-2	8 months	N/A
Living situation		
Alone	25 (15)	N/A
With Partner	132 (78)	N/A
With Children	16 (10)	N/A
Other	6 (4)	N/A

^a^Based on the first analysis of the PRESENT cohort by Van der Velden et al. [11]

None of the responders had been confirmatively infected by the SARS-CoV-2 virus at the time the questionnaire was returned (Table 2). Three responders were tested negative by nasopharyngeal swab. Eleven responders (7%) had experienced symptoms of fever and cough but were not tested for the virus. The majority of patients (85%) were worried to some extent about being infected by the COVID-19 virus. Eighty-three responders (49%) worried more about their (access to) health care due to the pandemic. When asked whether their global health status had changed since the start of the COVID-19 pandemic, 12 patients (7%) reported an improvement in their global health status, 43 (25%) reported a worsening, and 112 (65%) reported no change.

**Table 2:** Questions in the extra questionnaire sent out during lockdown about the effect of COVID-19 and the lockdown on the patient's health and health care

	Number of patients (N = 169)	Percentage
Are / were you infected by the COVID-19 virus?		
Yes, confirmed by nasopharyngeal swab	0	0.0
Possibly, I have or had fever and symptoms, but was not tested	11	6.5
No, I was tested negative	3	1.8
No, I had/ have no symptoms and I was not tested	148	87.6
Are you concerned about being infected by the COVID-19 virus?		
Not at all	17	10.1
A little bit	93	55.0
Quite a bit	45	26.6
Very much	6	3.6
Are you worried about your health care as a result of COVID-19?		
I worry more about my health care / treatment	83	49.1
I worry less about my health care / treatment	15	8.9
no difference since the start of COVID-19	64	37.9
Global patient impression of change regarding your global health status since start of COVID-19 lockdown		
Improved global health status	12	7.1
No change in global health status	112	66.3
Worsened global health status	43	25.4
Were there any changes in your health care as a result of COVID-19?		
No changes	52	30.8
Measures were taken but appointments were kept face to face	11	6.5
Hospital appointments were converted to online appointments	62	36.7
Hospital appointments were cancelled	25	14.8
Hospital appointments were postponed	17	10.1
My treatment was altered	15	8.9
My treatment was postponed	10	5.9
My treatment was cancelled	9	5.3
Second opinion was postponed	3	1.8
Second opinion was cancelled	1	0.6
Did the threshold to contact your general practitioner or oncologist change because of the COVID-19 situation?		
Yes, I contact my physician less easily	26	15.4
Yes, I contact my physician more easily	3	1.8
No, nothing changed in how easily I contact my physician	133	78.7
Did you leave your house the last few days?		
I don't go outside at all anymore due to COVID-19	28	16.6
I go outside rarely, for instance to go for a short walk	79	46.7
I go outside as much as I can taking social distancing into account	31	18.3
I go outside just as much as before COVID-19	10	5.9
Other	13	7.7
How were your social contacts (physical and virtual) the last few days?		
I have no or barely any contact with family and friends	23	13.6
Contact with my family and friends decreased	83	49.1
Contact with my family and friends stayed the same	37	21.9
I have more contact with my family and friends	18	11.1
Do you experience stress or anxiety due to the coronavirus?		
I am stressed or anxious due to COVID-19	47	27.8
I am not at all stressed or anxious due to COVID-19	49	29.1
There's no difference in my stress level due to COVID-19	65	38.5

Changes in health care trajectories were reported by 153 (84%) of the responders, most of which were appointments that were converted into online appointments, or appointments that were delayed or cancelled. Fifteen percent (n = 26) of the responders experienced a higher threshold to contact their treating physician or general practitioner due to the COVID-19 situation. Reasons were mostly linked to fear of overburdening health care professionals, or fear of having to enter the hospital during the pandemic.

With regard to stress and anxiety related to the coronavirus, 47 patients (28%) reported higher stress and anxiety levels due to the pandemic, 49 patients (29%) experienced no stress or anxiety whatsoever due to the pandemic, and 65 patients (39%) experienced stress and/or anxiety, but it had not increased due to COVID-19. Twenty-eight (17%) patients reported not to go outside at all due to lockdown measures, and 79 (49%) rarely went out. One hundred and six patients (63%) reported a decrease in physical and virtual contact with family and friends, twenty-three (14%) of whom reported to have barely any contact.

### Quality of life

Data on quality of life during lockdown were available from 201 patients. Information on the first timepoint before the start of lockdown (BL-1) was available for 178 patients (89%). For the second timepoint before start of lockdown (BL-2) information was available for 155 patients (77%) (Fig. 1).

**Fig. 2:**
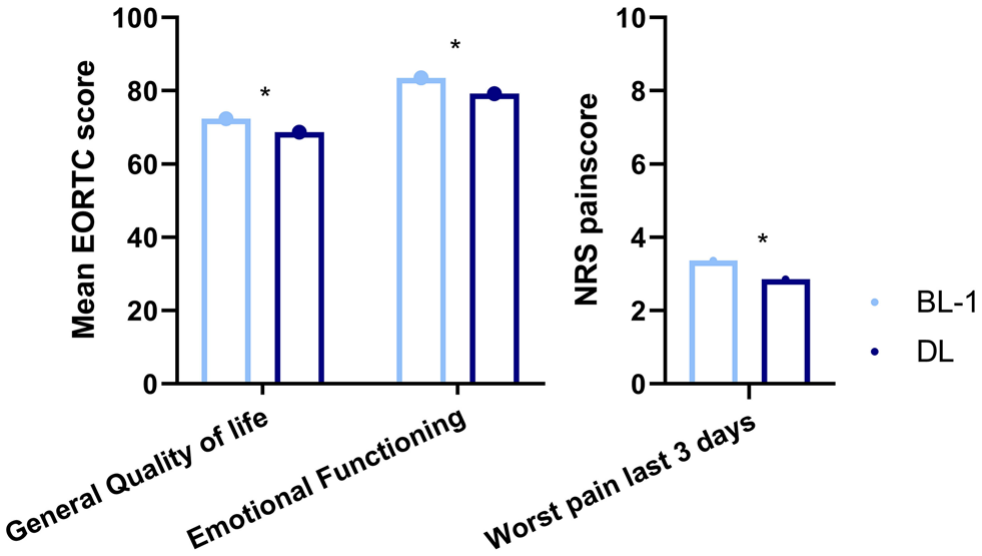
Barchart of mean EORTC scores of General Quality of Life and Emotional Functioning before and during lockdown. EORTC-QLQ C15 scores range from 0 to 100, higher scores represent better outcomes. Numeric rating scale (NRS) ranges from 0 to 10, higher scores represent more pain. *Statistically significant difference between the two timepoints

General quality of life was reported to be significantly lower during lockdown compared with BL-1 (Mean = 69 and 72 respectively, P = 0.007) (Fig. 2). This difference was no longer present when comparing quality of life during lockdown with timepoint BL-2. A significant difference was found between emotional functioning before and during the COVID-19 lockdown (with a mean of 84 and 79 respectively, P = 0.004) (Table 3). This difference was similar when comparing the scores with the BL-2 with the questionnaire filled out during lockdown (with a mean of of 85 and 80 respectively, P = 0.01).

**Table 3:** Paired *T*-test to compare quality of life domains before and during COVID-19 lockdown

Scale	Timepoint	n	Mean	SD	t	P
Worst pain 3 days (BPI)	During Lockdown	177	2.85	2.58	2.63	0.009
	Before Lockdown-1	177	3.36	2.82		
	During Lockdown	154	2.74	2.53	0.58	0.566
	Before Lockdown-2	154	2.86	2.73		
General Quality of Life (EORTC-C15-PAL)	During Lockdown	174	68.68	19.32	2.75	0.007
	Before Lockdown-1	174	72.41	18.77		
	During Lockdown	152	69.3	19.19	0.99	0.326
	Before Lockdown-2	152	71.05	20.37		
Emotional functioning scale (EORTC-C15-PAL)	During Lockdown	177	79.24	20.73	2.92	0.004
	Before Lockdown-1	177	83.57	18.55		
	During Lockdown	154	80.36	19.74	2.62	0.01
	Before Lockdown-2	154	84.80	18.20		

*BPI* Brief Pain Inventory, *EORTC* European Organization for Research and Treatment of Cancer, *SD* Standard diviation

^*^EORTC-QLQ C15 scores range from 0 to 100. Higher scores represent better outcomes

**Fig. 3:**
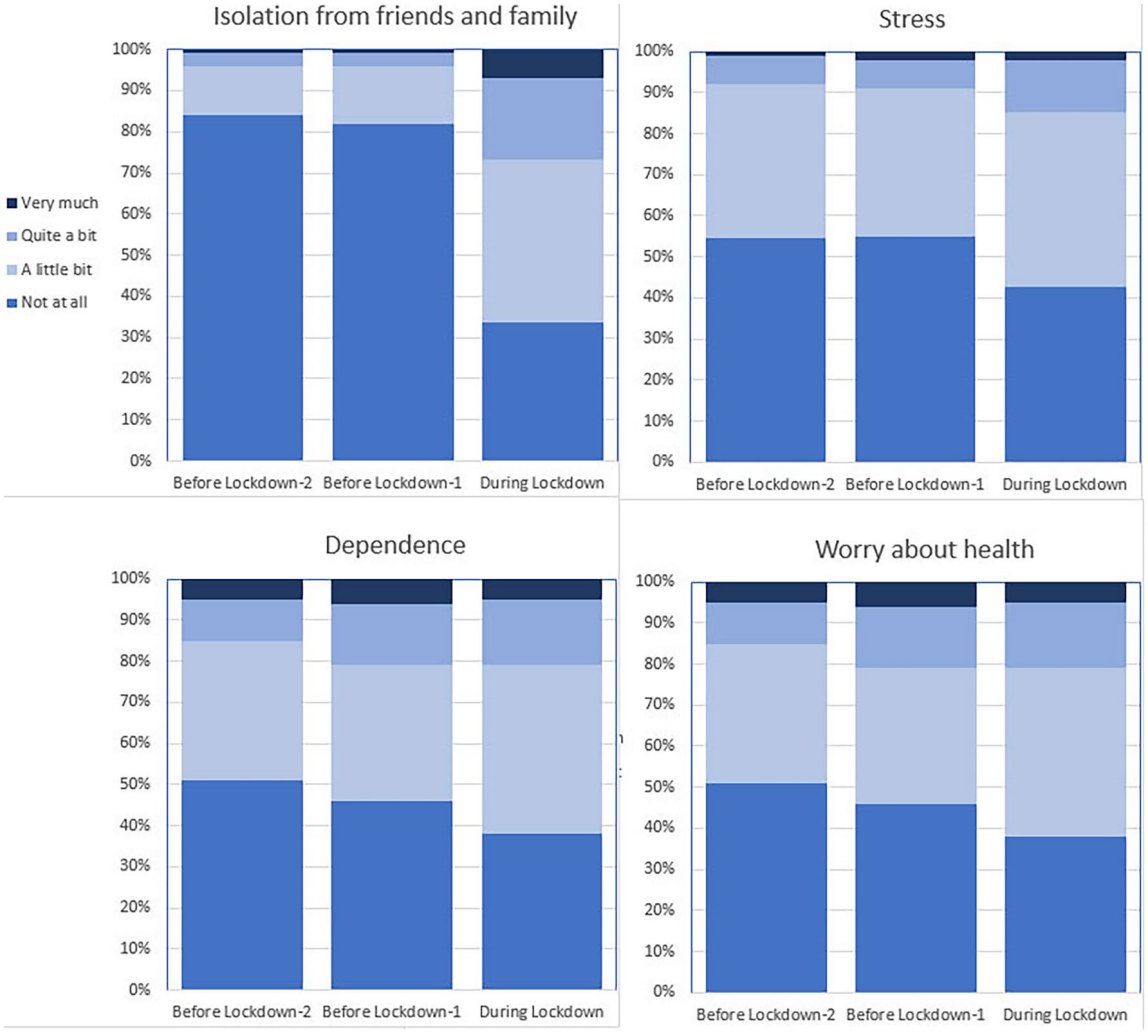
Changes in Quality of Life before and during the COVID-19 lockdown. Percentage of patients reporting complaints about feelings of isolation from friends and family, stress, dependence and worry about health on the timepoints before lockdown-2, before lockdown-1 and during lockdown. A steep increase in feelings of isolation is reported during lockdown

Reported pain scores (NRS 0–10) were lower during lockdown than BL-1 (Mean = 2.9 and 3.4 respectively, P = 0.009) (Table 3). Notable differences were reported for sense of isolation and stress. Before lockdown, 18% patients experienced some degree of isolation from close friends and relatives. During lockdown, this proportion increased to 67% of patients. Stress was reported to some extent by 45% of the patients before lockdown and by 58% of the patients during lockdown (Fig. 3 and Table 4).

## Discussion

General quality of life and emotional functioning of patients with metastatic bone disease deteriorated significantly during the COVID-19 lockdown in the Netherlands. There was a strong increase in feelings of isolation, from 18% before lockdown to 67% during lockdown, and 17% of the patients did not, or barely leave their homes during lockdown. The decrease of general quality of life and emotional functioning in these challenging times during the pandemic are worrying, even more so in this palliative population who may not get a chance to regain quality of life after the pandemic is over. Yang et al. have shown that loneliness is an important mediator between social isolation and cognitive functioning [15]. In other words, preventing loneliness can be very helpful to counteract the negative impact of social isolation on cognitive functioning. With the second wave we’re currently in, and the realistic prospect of a third wave of COVID-19 infections leading to another lockdown, it is suggested to be very important to start facilitating safe contact moments for patients and their (informal) care givers in order to prevent negative effects of isolation on quality of life. Online peer-to-peer contact or online mental health interventions might enhance emotional and psychosocial wellbeing during a continuing pandemic [16]. This could be in the form of organizing informational symposia that patients can join online or making sure that patients have the means to contact health care providers if the need arises.

**Table 4:** Frequencies of separate items in EORTC C15-PAL and BM22 questionnaires at two time points before start of lockdown and during lockdown

	Before lockdown-2 N (%)	Before lockdown-1 N (%)	During lockdown N (%)
Stress	N = 155	N = 178	N = 200
Not at all	84 (54)	98 (55)	85 (43)
A little bit	58 (37)	64 (36)	86 (43)
Quite a bit	11 (7)	13 (7)	26 (13)
Very much	2 (1)	3 (2)	3 (2)
Isolationfrom close friends and relatives	N = 153	N = 176	N = 197
Not at all	128 (84)	144 (82)	66 (34)
A little bit	19 (12)	25 (14)	78 (40)
Quite a bit	4 (3)	6 (3)	39 (20)
Very much	2 (1)	1 (1)	14 (7)
Dependence	N = 154	N = 176	N = 198
Not at all	79 (51)	81 (46)	75 (38)
A little bit	52 (34)	58 (33)	82 (41)
Quite a bit	16 (10)	27 (15)	32 (16)
Very much	7 (5)	10 (6)	9 (5)
Worry about health	N = 154	N = 176	N = 198
Not at all	30 (20)	42 (24)	29 (15)
A little bit	79 (51)	66 (38)	99 (50)
Quite a bit	30 (20)	51 (29)	55 (28)
Very much	15 (10)	17 (10)	15 (8)

Interestingly, reduced pain scores were reported during lockdown. This is a reassuring finding, as it shows that palliative treatment of pain remains adequate, even though many patients (89%) reported changes in their health care as a result of COVID-19, such as postponed or cancelled appointments and/or treatments. In previous studies, an association between pain response and quality of life was reported within the first three months after radiotherapy [17, 18]. In the current study we found an improvement of pain scores and a decrease in quality of life scores during the COVID-19 lockdown. This could indicate a decreasing association of pain response and quality of life in later stages of follow up, or a strong influence of COVID-19 measures on quality of life.

The prospective PRESENT cohort provided the unique opportunity to compare patient reported outcomes before the COVID-19 pandemic to outcomes during the pandemic in the same population. This study was timed during the acute phase of the COVID-19 pandemic, focusing on the short-term impact of COVID-19 measures on this patient population. However, it is known from publications on previous viral threats such as the H1N1 pandemic in 2009, that some psychological effects such as increased general anxiety can last up to 30 months after the H1N1 pandemic in 2009 [10].

One shortcoming of our study is that the COVID specific questionnaire was not available before COVID. Therefore, the reported answers to COVID specific questions are lacking a baseline comparison. Moreover, as a result of the COVID-19 measurements, active recruitment of new PRESENT-participants was temporarily suspended to minimize risk of transmission of the virus for this vulnerable patient population. Consequently, the conclusion of this study was predominantly based on patients who are no longer actively treated with radiotherapy for their painful bone metastases and could therefore be a selected population with a relatively long survival compared with other stage IV patients. Nevertheless, the study population was largely similar to the general PRESENT cohort with respect to baseline characteristics. The response rate to this study was also comparable with the response rate normally observed in the cohort [11]. Therefore, we expect our results to be representative of the general cohort population.

Although interpretation of single quality of life items may be limited when not used within the validated domain scales, we observed a notable increase in symptoms of isolation and stress. Feelings of isolation are to be expected and most likely linked to the imposed COVID-19 measures of lockdown, as patients were urged to stay home and have as little contact with other people as possible. Although these measures were important for containment of the virus, the effect they had on this specific population gives reason for concern. Given the nature of the prognosis of stage IV cancer, these patients cannot postpone their plans to pick up life when the pandemic is over. They may have missed out time for their personal wishes and activities before they are conditionally no longer able to do so. For this reason, the impact of social distancing could also be bigger in this population than in other (cancer) patients.

## Conclusion

This study has shown a strong increase in feelings of isolation and a decrease of emotional functioning and general quality of life during the COVID-19 lockdown in patients with stage IV cancer who were treated in our institution for painful bone metastases. Whilst COVID-19 measures are important to control and reduce further spread of the Sars-CoV-2 virus, the implications of these measures on vulnerable populations should not be overlooked. The increased feelings of isolation and psychological stress should be minimized by creating safe contact moments for patients and their support network.

## Data Availability

Research data are stored in an institutional repository and will be shared upon reasonable request to the corresponding author.

## Abbreviations

PROs: Patient reported outcomes
PRESENT: Prospective evaluation of interventional StudiEs on boNe meTastases
UMCU: University Medical Center Utrecht
BPI: Brief pain inventory
EORTC: European Organisation for Research and Treatment of Cancer
DL: During lockdown—timpoint during lockdown
BL-1: Before lockdown 1—first timepoint before start of lockdown
BL-2: Before lockdown 2—second timepoint before start of lockdown
M: Mean
SD: Standard deviation
IQR: Inter quartile range
SPSS: Statistical Package for Social Sciences
